# Polio-(Trepho)-Myelitis Anterior

**Published:** 1883-03

**Authors:** 


### Extracts.

Polio-(Trepho)-Myelitis Anterior.—The rarity of this disease, and its peculiar symptoms, have always interested physicians. The following case will show symptoms which are not always observed. A physician, forty-one years of age, and a very busy practitioner, felt suddenly a weakness in the lower extremities. During tne day he was able to walk, but on the same evening he could not climb the stairs without being assisted. At first, the muscles of the upper part of the left limb were affected, and then the muscles of the right limb. Paresis of the lower extremities followed during the next two days, and on the fifth day paralysis of the same. At the same time, a weakness of the muscles in the dorsal region was noticed; then paresis and paralysis of the right and left arms, and at last the muscles of the neck and face were attacked. On the eleventh day, the *status præsens* was as follows: The patient is of a gracile structure, without panniculus adiposus; the lower extremities are paralyzed; the muscles lax; passive motion not impaired; perfect paralysis of the muscles of the middle part of the body; not the slightest motion can be executed by this part. Left arm motionless; on the right arm only the fingers can be moved. Paresis of the muscles of the face, but the mouth can be opened; the patient cannot spit out. The eyes can be closed and opened; knitting the brows is impossible, also deep inspiration and expectoration. With the exception of slight movements of the head and the right hand, the body is motionless. Sphincters free. Urination but seldom; erine without albumen. Defection must be brought on by high injections. The sensibility of the lower extremities is abolished; the sense of touch is lost; the patient cannot discern if a piece of wood or cloth is laid between the toes or fingers. Temperature and the sensation of pain very much diminished. The reflex sensation of the skin and the tendons is abolished. Pupils small, but they react normally. The patient is morally depressed, believing that he suffers from Gaudry's paralysis, and that he must die. After three weeks, decubitus set in, but after four to six weeks' treatment with the Faradaic current, the patient recovered entirely. There were some symptoms of syphilitic affection, and the patient had had syphilis twenty years ago, but the treatment proved the true nature of the disease.—*St. Petersburg Med. Wochenschrift.*

